# Lessons learned from a multi-centre implementation of an artificial intelligence algorithm to detect vertebral fractures for radiology, information technology, information governance and clinical leads

**DOI:** 10.1093/bjrai/ubaf017

**Published:** 2025-10-24

**Authors:** Rohit Vijjhalwar, Kaiyang Song, David D G Chappell, Jane Turton, Jack Boylan, Jane Threlkeld, Mike Page, Madeleine Sampson, Yuriy Arlachov, Cass Chisholm, Rachel Eckert, Raz Edwards, Amrita Kumar, Elizabeth Curtis, Kenneth E S Poole, Yotam Kimmel, Oren Shalem, Michael Stone, Rafael Pinedo-Villanueva, Opinder Sahota, Muhammad Kassim Javaid

**Affiliations:** Translational and Experimental Sciences Division, University Hospital Southampton NHS Foundation Trust, Southampton, SO16 6YD, United Kingdom; Department of Rheumatology, Guy’s and St Thomas’ NHS Foundation Trust, London, SE11 4TX, United Kingdom; Department of Medicine, University of Cambridge, NIHR Cambridge Biomedical Research Centre, Cambridge, CB2 0QQ, United Kingdom; Geriatric Medicine and General Internal Medicine, Cardiff and Vale University Health Board, Cardiff, CF11 4XW, United Kingdom; Geriatric Medicine and General Internal Medicine, Cardiff and Vale University Health Board, Cardiff, CF11 4XW, United Kingdom; School of Medicine, Cardiff University, Cardiff, CF14 4YS, United Kingdom; Department of Radiology, Bradford Teaching Hospitals NHS Foundation Trust, Bradford, BD9 6RJ, United Kingdom; Department of Radiology, Bradford Teaching Hospitals NHS Foundation Trust, Bradford, BD9 6RJ, United Kingdom; Translational and Experimental Sciences Division, University Hospital Southampton NHS Foundation Trust, Southampton, SO16 6YD, United Kingdom; Department of Radiology, Nottingham University Hospitals NHS Trust, Nottingham, NG5 1PB, United Kingdom; Department of Radiology, Bradford Teaching Hospitals NHS Foundation Trust, Bradford, BD9 6RJ, United Kingdom; Fracture Prevention Service, Oxford University Hospitals NHS Foundation Trust, Oxford, OX3 7HE, United Kingdom; Data Security and Protection, The Royal Wolverhampton NHS Trust, Wolverhampton, WV1 9TD, United Kingdom; Department of Radiology, Frimley Health NHS Foundation Trust, Frimley, GU16 7UJ, United Kingdom; MRC Lifecourse Epidemiology Centre, University of Southampton, Southampton, SO16 6YD, United Kingdom; Department of Medicine, University of Cambridge, NIHR Cambridge Biomedical Research Centre, Cambridge, CB2 0QQ, United Kingdom; Nanox AI, Petah Tikva, 4970602, Israel; Nanox AI, Petah Tikva, 4970602, Israel; Geriatric Medicine and General Internal Medicine, Cardiff and Vale University Health Board, Cardiff, CF11 4XW, United Kingdom; School of Medicine, Cardiff University, Cardiff, CF14 4YS, United Kingdom; Nuffield Department of Orthopedics, Rheumatology and Musculoskeletal Sciences, University of Oxford, Oxford, OX3 7LD, United Kingdom; NIHR Oxford Biomedical Research Centre, Oxford, OX3 7LD, United Kingdom; Department of Health Care of Older People, Nottingham University Hospitals NHS Trust, Nottingham, NG5 1PB, United Kingdom; Nuffield Department of Orthopedics, Rheumatology and Musculoskeletal Sciences, University of Oxford, Oxford, OX3 7LD, United Kingdom; NIHR Oxford Biomedical Research Centre, Oxford, OX3 7LD, United Kingdom

**Keywords:** osteoporosis, artificial intelligence, spinal fractures, fracture liaison service, information governance, information technology

## Abstract

Artificial intelligence (AI) algorithms have been developed to identify vertebral fractures through reanalysis of existing CT scans. This study describes a real-world case study of the deployment of an AI solution in the NHS, from information governance (IG) and technology (IT) perspectives, to inform best practice recommendations. Five NHS hospitals were selected to deploy the Nanox AI solution to identify vertebral fractures from existing CT images. The journey to IG and IT assurance was described and used to inform recommendations. The time from contract signing to IG assurance ranged between 5 and 13 months. The period from IG assurance to the analysis of the first patient scan ranged from 7 to 12 months, excluding 1 site withdrawing from the process. Each site required different IG documents: Data Protection Impact Assessment (5/5 sites), Data Protection Agreement (2/5 sites), Digital Technology Assessment Criteria (2/5 sites). IT implementation delays included third-party supplier coordination, NHS IT staff availability, and local capability. Based on the observed challenges, 6 best practice recommendations are proposed to address current challenges to AI adoption in radiology settings in the NHS to support IG and 8 to support IT implementation services. Significant challenges remain if AI is to be routinely used to identify vertebral fractures. The proposed recommendations provide a pathway to improve effective and efficient AI deployment. This study proposes recommendations from IG and IT perspectives to improve the local deployment of AI in the NHS.

## Introduction

As the population ages and the prevalence of co-morbidities increases,^1^ the demand for radiological scans in the United Kingdom has been rising at a rate disproportionate to the number of available radiologists.^2^ This growing demand has placed significant pressure on radiologists to report scans promptly.^3–5^ Integrating artificial intelligence (AI) tools into radiology can revolutionize clinical practice by enhancing diagnostic accuracy, personalizing treatment, and improving patient outcomes and the efficiency of the healthcare system. Artificial intelligence-supported software solutions are increasingly matching or even surpassing human experts in specific tasks, with recent studies showing that AI-augmented radiologist assessments achieve higher accuracy in image classification compared to AI or radiologists alone.^6–8^ In addition, introducing AI platforms into hospital radiology workflows can lead to substantial benefits, including time savings for radiologists and staff, highlighting its potential to streamline operations and improve efficiency.^9^

Despite these benefits, the integration of AI technologies into routine NHS radiology workflows presents significant challenges, including regulatory compliance and technology (IT) requirements, with a call for more real-world evidence to support equitable implementation.^10^ Currently, most research has focused on the clinical performance of AI models with little research on the IT/IG challenges.

The principles of implementation science focus on how innovations can be effectively integrated into existing health systems.^11^ In radiology, this involves navigating complex site-level processes including data protection, clinical safety, technical compatibility, and local regulatory compliance. Understanding these processes is essential for Picture Archiving and Communication System (PACS) managers, R&D leads, IT and IG teams and NHS digital transformation programmes.

Artificial intelligence systems used in direct clinical care must also meet clinical safety requirements. NHS Digital mandates that such systems comply with clinical risk management for developers (DCB0129) and deploying health organizations (DCB0160).^12^ These standards require safety cases and hazard logs to ensure clinical risks are clearly identified, documented, and managed.

Specifically, within the United Kingdom, the regulatory landscape has evolved significantly over the past decade. The 2019 Topol Review emphasised the need to improve digital literacy of the NHS workforce to realize improvements in patient outcomes from adoption of AI-based technologies, including data governance, assessment, and commissioning.^13^ The 2020 Caldicott Principles established 8 key standards for sharing patient information for individual and for other purposes.^14^ The 2021 NHS Digital Technology Assessment Criteria (DTAC) provides common baseline criteria for healthcare organizations to assess the clinical safety, data protection, cyber security, interoperability, and accessibility of digital health tools at the point of procurement.^15^ The 2025 10-year Health Plan for England set a strategic direction for technology adoption including an AI-enabled NHS as part of treatment to improve clinical outcomes, focusing on switching from analogue to digital as core to delivering both national and neighbourhood health service improvements.^16^ Artificial intelligence tools that process imaging data typically require access to patient-identifiable information. In many cases, this includes cloud-based processing. These scenarios are considered high risk under the UK General Data Protection Regulation (GDPR) and require a Data Protection Impact Assessment (DPIA). The DPIA identifies potential privacy risks and sets out mitigation strategies including lawful basis, data minimization, retention periods, and transparency measures. NHS England provides a national template to support this process.^17^ Hence, together, the DPIA and DCB standards form the foundation of safe and lawful AI deployment in NHS settings.

Alongside regulation, the successful deployment of AI solutions in the NHS radiology departments is also shaped by substantial variability in NHS IT infrastructure. Some Trusts operate in-house PACS and EHR systems, while others rely on third-party providers that limit local control. This affected technical capabilities such as configuring DICOM routing, setting up servers, and managing secure outbound connections. These differences contribute to variation in deployment timescales, costs, and staffing requirements.

Although national frameworks such as the Royal College of Radiologists’ 2024 guidance AI Deployment Fundamentals for Medical Imaging are now available, there remains limited detailed practical guidance for IG assurance and IT implementation of AI-based technologies for NHS Trusts.^18^ Most published studies focus on technical performance rather than operational barriers. This study provides a multi-site case study of the deployment of this AI tool from IG and IT perspectives in 5 NHS Trusts to inform the best practice recommendations for each of the key stakeholders.

This study focuses on the integration of an AI tool into NHS radiology systems, using vertebral fracture detection as a use case. While the tool facilitates onward referral to Fracture Liaison Services, the study examines the technical, governance, and operational processes required for AI implementation within radiology infrastructure. This study aims to support hospital managers, IG/IT teams, R&D staff, and clinical leads by offering actionable insights to facilitate safe and effective AI implementation.

## Methods

Nanox AI HealthVCF solution^19^ is a CE-marked class IIa medical device that automatically identifies vertebral compression fractures (VCFs) on routine CT scans that include the thoracic or lumbar spine.^20^ The HealthVCF identifies VCFs by identifying vertebrae with a reduction in vertebral body height of more than 25%, in accordance with Genant’s classification of moderate and severe fractures.^20^^,^^21^ If a vertebral fracture is detected, it sends alerts to a clinical workstation for validation by a locally trained clinician either directly to the facility PACS (hospital A) or via the Nanox REAL platform for asynchronous analysis (hospital B-E). Once a VCF was confirmed, a short code was added to the report that generated an automatic referral to the FLS pathway in hospital A that used augmented radiology reporting. For the asynchronous analysis, the local FLS could export a spreadsheet of the patient and scan details for all positively confirmed scans. Where the AI-enabled vertebral fractures were identified but not mentioned in the routine radiology report, that is, a discrepancy, only hospital B routinely reports the findings into their local Radiology Events and Learning Meeting.

**Figure 1.**
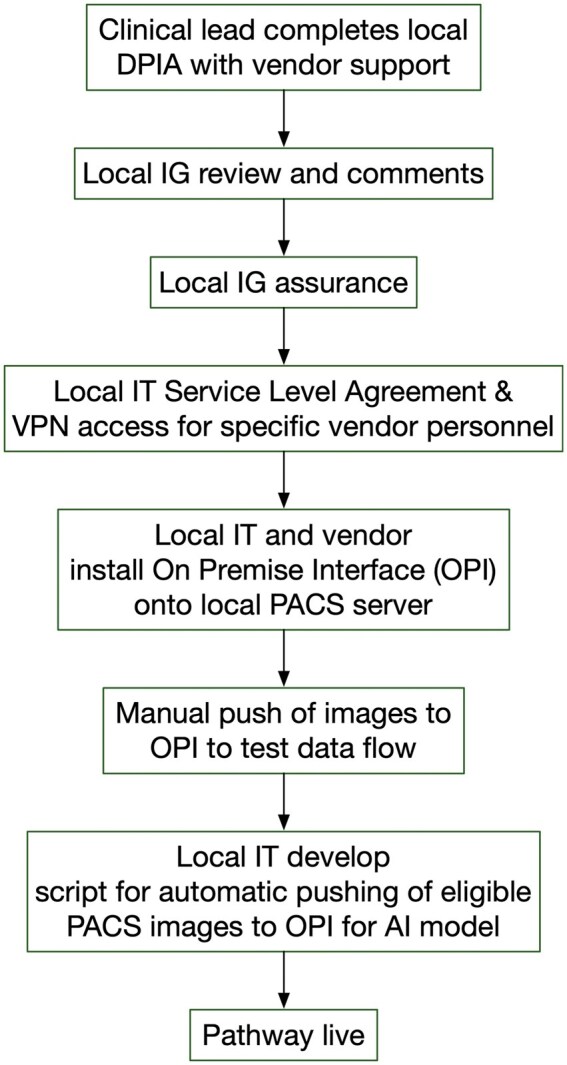
Original Oxford implementation experience. Abbreviations: AI = artificial intelligence; DPIA = Data Protection Impact Assessment; IG = information governance; OPI = On Premise Interface; PACS = Picture Archiving and Communication System; VPN = Virtual Private Network.

**Figure 2.**
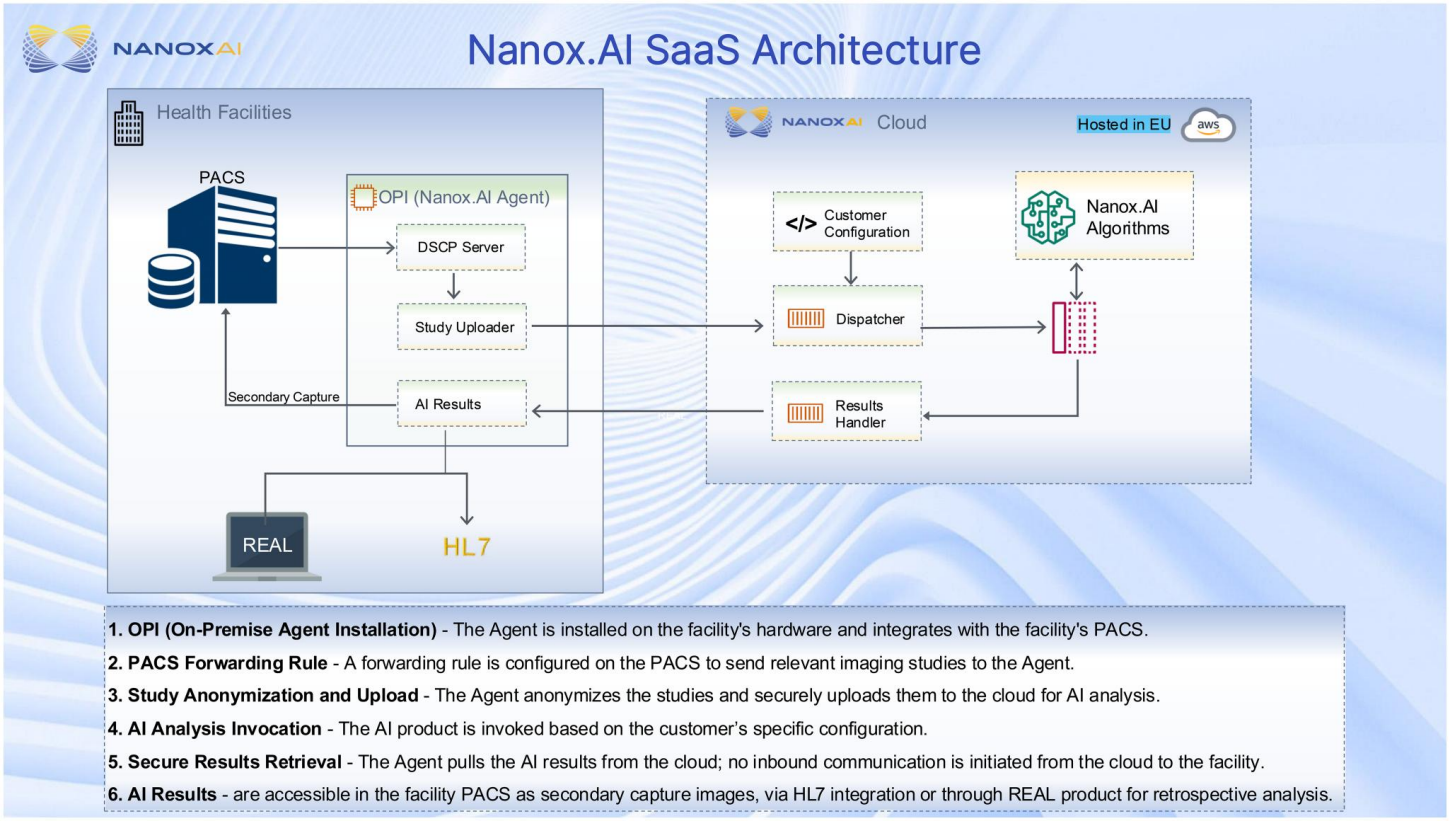
Data flow architecture of the Nanox.AI HealthVCF system for vertebral fracture detection. Abbreviations: AI = artificial intelligence; DSCP = Differentiated Services Code Point; PACS = Picture Archiving and Communication System.

Deployment of HealthVCF requires integration with local radiology IT infrastructure. This includes a dedicated on-premises server, configuration of automatic image forwarding rules from the PACS, and secure HTTPS communication with cloud infrastructure. While the AI tool processes data on a server located in Ireland (within the EU data protection framework), auxiliary functions such as software updates, remote monitoring, and system maintenance are hosted via Amazon Web Services (AWS) in the United States and require encrypted outbound internet access (HTTPS on port 443). A web-based viewer component, REAL, allows radiologists to review flagged results, loading interface elements dynamically from the cloud upon login. Although no patient-identifiable data leaves the NHS Trust network and all communications are encrypted using modern TLS protocols, these technical requirements must be reviewed and approved by each Trust’s Information Governance (IG) and IT teams. Figure 1 outlines the typical implementation workflow based on the mentor site experience. Figure 2 depicts the data flow architecture from PACS to the AI server and back to the clinical viewer.

Nanox AI employed a robust set of cloud security measures to ensure patient data protection and compliance with relevant regulations. All scans were anonymized on-site using the HIPAA Harbor method, removing all 18 protected health identifiers before transmission to the cloud. Access to cloud services was restricted via IP whitelisting, and all data was encrypted both in transit and at rest using TLS v1.2. Secure access control was enforced through token-based OAuth2 authentication. To monitor and mitigate risks, AWS-native tools such as GuardDuty and Inspector were used for anomaly detection and vulnerability assessment. High availability was ensured through multi-availability zone deployment, supporting system resilience and uptime. Continuous monitoring and audit logging were implemented to maintain traceability and compliance.

Customer data, including monitoring information and usage metrics, were stored exclusively in AWS EU regions. No AI analysis data were permanently retained on the cloud. Production and test environments were fully separated to prevent R&D access to operational data. Additionally, customer support staff were trained not to request or disclose any identifiable patient information during support interactions.

The HealthVCF tool was part of the NHSX and National Institute for Health Research–funded multi-centre AI-enabled Detection of Osteoporosis for Treatment (ADOPT) study (AI_AWARD02169) evaluating the patient and economic outcomes from AI-enabled detection of vertebral fractures in the FLS setting in 2021. Oxford was the mentor site given its experience in FLS since 2008 and use of HealthVCF since 2018. Five hospitals were selected from across the country representing the 5 major regions across England and Wales for the implementation of the Nanox AI tool, with a view to improving patient outcomes for existing FLSs. All sites had existing FLSs and were actively participating with the national mandatory HQIP FLS Database (FLS-DB).^22^ This included large teaching hospitals and a district general hospital. Implementation and evaluation sites were selected based on expected caseload, hospital setting (ie, teaching hospital vs district general hospital), and current performance of the FLS using the FLS-DB. Subsequently, a site-level detailed FLS questionnaire was sent to candidate sites to evaluate the FLS organization structure, existing capacity, funding status, and expected opportunities and barriers for implementing the AI-enhanced pathway.

A lead clinician in each hospital was supported to work with Nanox AI and the University of Oxford to secure both IG assurance and IT support. In hospital A, the lead clinician was the musculoskeletal radiology lead; in hospital B-E, the lead clinician was the local metabolic bone lead. The lead clinician in hospital D had experience in AI implementation. The lead clinician at each site was involved in the initial grant application and their time was covered by the grant to submit the local regulatory materials based on existing mentor site documents and experience.

The IG and IT processes were modelled on the approval process from the mentor site including who the key local stakeholders are and an example of their DPIA document (Figure 1). The mentor site shared these resources with the clinical lead who was delegated to lead the process locally, including identifying other local stakeholders, additional regulatory processes and forms, localising forms, arranging their submission, and responding to queries. Initially, only the local clinical leads were supported regarding the IG and IT processes based on the pathway used at the mentor site. The mentor team also provided site-specific implementation support, including a copy of their completed DPIA, example documentation, and a detailed process map (Table S1). These materials were used by local IG and IT teams as a reference and adapted to meet individual Trust requirements. Sites were encouraged to follow the same approval stages, but local customization was required for documentation, stakeholder engagement, and internal reviews. However, the local sites were requesting additional documents for which there were no mentor templates to follow that led to the mentor site team directly communicating with the local IG teams.

Data on the implementation process were gathered using a mixed qualitative and operational approach. Information was drawn from site-level questionnaires collected during the setup phase, records of weekly multi-site implementation meetings, email correspondence, submission timelines, and IG documentation. These records included formal and informal communications between local teams, the mentor site, and the vendor. All data were collected during the course of the project to support delivery but were retrospectively analysed for the purposes of this study. There was no prospective plan to measure the time taken to secure IG approvals, catalogue the forms submitted or evaluate response delays.

## Results

### Site characteristics and pathway variation

Among the 5 selected sites, 4 were large-volume teaching hospitals and 1 medium-volume district general hospital (Table 1). Three hospitals had a partial vertebral fracture pathway, while the remaining 2 sites did not have a routine vertebral fracture pathway. Where the AI-enabled vertebral fractures were identified but not mentioned in the routine radiology report, that is, a discrepancy, only hospital B routinely report the findings into their local Radiology Events and Learning Meeting. The interval from the project start (defined as when the contract was signed) to the IG agreement varied from 5 to 13 months. The time from the IG agreement to the first patient scan analysed ranged from 7 to 13 months, with one site withdrawing (Figure S1).

**Table 1.** Type of hospital, current spine fracture pathway status, lead clinician occupation, prior experience in AI implementation and time to approval across each hospital.

Timelines	Hospital A	Hospital B	Hospital C	Hospital D	Hospital E
Type of hospital	Medium-volume teaching hospital	Large-volume teaching hospital	Medium-volume district general hospital	Large-volume teaching hospital	Large-volume teaching hospital
Lead clinician specialty	Radiologist	Radiologist	Metabolic bone consultant	Radiologist	Radiologist Trainee
Prior experience in implementation of AI solutions	No	No	No	Yes	No
Current spine fracture pathway status	Partial pathway present	No routine pathway	No routine present	Partial pathway present	Partial pathway present
Date of collaboration agreement	03/23^a^	04/22	04/22	04/22	04/22
Date of DPIA sign-off	05/23	09/22	03/23	05/23	10/22
Time from collaboration sign-off to local DPIA approval (months)	13^a^	5	11	13	6
Date of first patient forwarded and analysed	12/23	09/23	12/23	TBD	11/23
Time from IG approval to first patient analysed (months)	7	12	9	>8	13

a Hospital A did not sign the collaborative agreement until all documents were ready and so the time from the collaborative agreement to DPIA was set from April 2022. Abbreviation: AI = artificial intelligence; DPIA = Data Protection Impact Assessment; IG = information governance; TBD = To be determined.

### IG processes and delays

For each site, there was only 1 designated contact for the IG with often no cover during periods of annual leave or sickness. Further, the remit of the IG assurance ranged from issues related to the handling of patient data to wider issues related to funding for the AI, clinical risk, and interface with the research component of the project (Table 2). At hospital D, IG responses were initially focused on the interface between clinical service delivery and research. This was resolved after 12 months of correspondence, involving the local Trust head of Research. Despite this resolution, the IG team raised further concerns regarding indemnity and procurement that were not raised by other sites, as well as uncertainties related to timelines for IT implementation. Consequently, it was mutually agreed that hospital D would withdraw from the project. In hospital E, issues around clinical liability were raised which led to the study being registered by the mentoring team on the local NHS Resolutions risk register and resolved outside of the IG process.

**Table 2.** Information governance (IG) forms required by each hospital site.

Forms required	Hospital A	Hospital B	Hospital C	Hospital D	Hospital E	Total hospitals requiring form
DPIA	X	X	X	X	X	5 (100%)
DPA			X	X		2 (40%)
DTAC		X			X	2 (40%)
IG checklist application		X				1 (20%)
Clinical risk management plan		X			X	2 (40%)
Clinical risk management system		X			X	2 (40%)
Clinical safety case report		X			X	2 (40%)
Clinical safety hazard log		X			X	2 (40%)
System-level security template		X				1 (20%)
Third-party checklist contractor		X				1 (20%)
Cloud computing risk assessment			X			1 (20%)
Firewall request form			X			1 (20%)
Information governance DSPT toolkit			X			1 (20%)
International Data transfer agreement			X			1 (20%)
Due diligence form				X		1 (20%)
Additional services request				X		1 (20%)
Caldicott approval form					X	1 (20%)
Total forms required per hospital	1 (6%)	9 (50%)	6 (33%)	3 (17%)	6 (33%)	

Abbreviations: DPA = Data Processing Agreement; DPIA = Data Protection Impact Assessment; DSPT = Data Security and Protection Toolkit; DTAC = NHS Digital Technology Assessment Criteria.

### DPIA completion and content variation

Each site required a unique set of IG documents which included at least the DPIA (Table 2). The NHS provide a template DPIA form, with 11 distinct sections, summarized in Table 3. However, the contents of the forms varied between sites. Hospital A’s DPIA did not address key domains including whether the intended use of data is lawful (section 5 of NHS England DPIA template), how long the data are being kept and what will happen to it after that time (section 7), how people’s rights and choices are being met (section 8).

**Table 3.** Comparison of the contents of the NHS England template DPIA form and the DPIA forms from selected hospitals.

Section	Hospital A	Hospital B	Hospital C^a^	Hospital D	Hospital E
Screening question for whether DPIA form is required	Flow diagram	10-item screening questionnaire	9-item screening questionnaire	Not applicable	8-item screening questionnaire
Purpose of data use	Basic project aim questions and processing methods	Purpose and aim of data collection	Purpose and aim of data collection	Purpose and benefits of data collection and implementation of tool	Purpose and aim of data collection
What data do you want to use/share?	Tick-box options for data types	Free-text for data types being used/shared	Detailed data field descriptions and tick-box options for data types	Free-text for data types being used/shared and justification for each	Personal data listing
Where will data flow?	Narrative description	Narrative description	Narrative with diagram (diagram preferable)	Flow map required only. No narrative description required	Narrative description to include list of third-party organizations and partners involved
Is the intended use of data lawful?	–	Detailed legal basis questions	Single choice from a tick box for the legal basis of this data collection/sharing	Single choice from a tick box for the legal basis of this data collection/sharing	Detailed legal basis questions
How are you keeping the data secure?	Listing general risk reduction actions	Multiple security procedure questions around sharing and collecting data	Narrative description of security controls and access management	Technical controls and encryption type to protect data while sharing and storing	Technical questions about security, access and compliance
How long is the data retained?	–	Retention period length and anonymization descriptions	Retention and disposal plan description	Data end-of-use procedure description	Retention period length and manual/automatic deletion option descriptions
How are people’s rights and choices being met?	–	Data access requests procedure descriptions	Data access requests procedure descriptions	Data access requests procedure descriptions and which parts of data will/can be shared	How patients are informed about data use description
Which organizations are involved?	General stakeholder consultation	Detailed stakeholder listing	Detailed stakeholder list with impact	Data access control with stakeholder measures	Third-party sharing procedures
Risk assessment with regards to data protection	Privacy issues listing but with no scoring	Privacy issues listing but with scoring and a mitigation table	Privacy issues listing but with scoring and a mitigation table	Privacy issues listing but with scoring and a mitigation table	Privacy issues listing but with no scoring and a mitigation table
Approval and sign-off	Single approval required—did not specify who	Single approval required—did not specify who	Single approval required—did not specify who	Multiple roles required for sign-off including Caldicott guardian, SIRO, data protection officer and Divisional information assets owner	Single approval require by Head of Data protection

a Hospital C is located within a devolved nation, but a national NHS DPIA form could not be found. Therefore, the DPIA form of hospital of hospital C is compared with the NHS England DPIA form.

Across the remaining 4 sites, the respective DPIA forms aligned with the NHS DPIA template by requiring information relating to why the data are needed (section 2 of DPIA), what data they wanted to use or share (section 3), and where data will flow (section 4), how long the data are being kept for and what happens to the data after that time (section 7), how people’s right and choices are being met (section 8), which organizations are involved (section 9). Across these 4 sites, the level of detail and clarification required across these sections differed with additional information such as data anonymization strategies and specific data handling procedures being required by the DPIA form at hospital A. All 5 sites’ DPIA forms included a risk assessment table, typically outlining risks to individuals, compliance risks, and organization/corporate risks, though hospital A was the only site not to use a risk scoring table.

### Heterogeneity in other IG documentation required

Aside from the DPIA forms, there was heterogeneity in the other IG forms that were required across the 5 sites. Hospital A solely required the DPIA form. Including the DPIA form, hospital B required 9 forms, hospital C required 6, hospital D required 4, and hospital E required 7 (Table 2). Certain IG documents (eg, system-level security template, third-party contractor checklist, firewall request form, information security toolkit, International Data Transfer Agreement, Due diligence form, Additional services request, Caldicott approval form) were only required by one of the selected sites. Both hospitals B and E also required the clinical safety and clinical risk management forms, IG checklist form, and DTAC document.

In addition, some Trusts required detailed data deletion and retention policies up front, while others focused more heavily on network security and firewall rules. Coordination was often needed between local teams, vendor representatives, and national IG advisors to align these expectations. This variation in practice highlighted the lack of a unified national standard and led to the formulation of structured recommendations based on the challenges encountered (see Table 4).

**Table 4.** Recommendations for improving implementation of AI in the NHS setting from an IG and IT setting.

Recommendations for IG approval pathway	Responsible stakeholders
1. We recommend that local IG departments estimate and communicate anticipated resource and cost requirements to clinical teams before AI procurement to support planning and sustainability.	Local IG director/team
2. We recommend that each Trust develop a standardized IG governance overview, outlining scope, responsibilities, key processes, and escalation mechanisms both internally and externally.	Local IG director/team
3. We recommend that AI providers should supply a comprehensive data flow map covering the end-to-end journey of both identifiable and de-identified data, including de-identification, re-identification, retention, and destruction protocols.	AI provider with local clinician lead
4. We recommend that a joint project initiation meeting should be held to clarify project scope, documentation requirements, approval timelines, and stakeholder roles.	Local clinical lead, local IG team, AI provider
5. We recommend a collaborative approach to completing IG documentation, with AI providers supporting Trusts in aligning responses with national DPIA and DTAC templates.	AI provider, IG team, local clinical lead
6. We recommend that clarification queries be formally tracked and, where needed, resolved via additional stakeholder meetings to reduce email-based fragmentation and delays.	Local clinical lead, AI provider, IG team

**Recommendations for IT implementation pathway**	

1. We recommend that anticipated IT support costs and third-party fees are communicated to clinical leads at an early stage to ensure feasibility and budget alignment.	Local IT department
2. We recommend that IT departments develop and share a written framework outlining scope, responsibilities, escalation processes and points of contact for AI deployment support.	Local IT department
3. We recommend AI providers provide detailed pre-installation specifications including hardware, software, PACS configurations, VPN needs, and uptime/maintenance access requirements to avoid downstream delays.	AI provider
4. We recommend that both parties should have a structured technical kick-off meeting to agree milestones, responsibilities, server setup tasks and firewall or VPN needs.	Local clinical lead, digital team PMO, AI provider, PACS manager, clinical safety officer Digital team PMO with AI provider support
5. We recommend the usage of project management tools to prevent overlooking approvals which track the completion of essential IT forms (e.g. VPN access, SLA agreements).	Digital team PMO, PACS team, AI provider, local clinical lead
6. We recommend for stakeholders to proactively schedule check-in meetings to resolve IT queries, instead of relying solely on asynchronous communications.	Digital team PMO, clinical safety officer, AI provider
7. We recommend for the use of an AGILE project delivery model, with defined backlogs, sprint cycles and regular technical meetings until system readiness is achieved.	Digital team PMO, clinical safety officer, AI provider
8. We recommend mandatory shadow testing before go-live, including imaging forwarding, system access, AI analysis return, and interface checks.	PACS manager, AI vendor, local clinical lead

Abbreviations: AI = artificial intelligence; DPIA = Data Protection Impact Assessment; DTAC = NHS Digital Technology Assessment Criteria; IG = information governance; PACS = Picture Archiving and Communication System; PMO = Project Management Office; VPN = Virtual Private Network.

### IT readiness and pre-installation challenges

A standard pre-installation information technology (IT) form (Table S1) was sent to all sites for completion, with the intention for the IG and IT processes to be completed in parallel as funding and collaborative contract for the project had already been agreed. However, 1 hospital could not initiate any IT activities until IG approval was granted. IT delays were due to various reasons including working with third-party suppliers, the availability of NHS IT staff, and local capability to generate automatic forwarding rules. We had estimated £6110 for the server space and setup. In hospital A, the local PACS IT lead configured the server and forwarding rules in preparation for the IG assurance at no additional cost. In hospitals B and C, a third-party supplier is responsible for delivering the local radiology services which led to significant delays. In hospital B, this led to a delay until £20 000 for the IT support had been received by the third-party supplier. In hospital C, the cost was £15 000 for the IT support but there were delays of more than 4 weeks in arranging meetings and responding to queries. Figure S1 provides a summary of costs to the hospitals.

#### Delays due to local IT constraints

One of the most persistent delays across the sites is related to the availability and capacity of local IT teams. This was the case even in hospitals where radiology services were managed internally rather than by external contractors. At hospitals D and E, there were automatic delays of 4 and 6 months, respectively, between submitting the initial IT setup request and receiving a response. These delays occurred despite funding already being secured, and they affected basic tasks such as reviewing the IT installation requirements and completing a standard setup checklist.

### Delays due to scan forwarding configuration and technical issues

A key technical requirement of the Nanox AI platform is the ability to forward relevant CT scans, specifically those including the thoracic or lumbar spine, from the hospital’s PACS to an on-premises server where the AI software is installed. This is typically done by configuring an automatic image routing rule, also known as a DICOM forwarding rule. This rule filters out eligible studies and sends them directly to the AI server for processing without requiring manual action. Hospitals A, B, and C were able to configure this successfully. However, in hospital E, local IT constraints meant that the PACS lead was initially unable to create this automated rule. Instead, images were transferred manually once per week. This workaround introduced delays, added staff burden, and created inconsistency in scan availability for AI analysis. The IT team at hospital E eventually received support to configure a working automated forwarding process, but this required additional time and training.

In hospital B, where radiology was delivered by a third-party service provider, the automatic forwarding rule caused a critical error during setup. Instead of sending a single copy of a CT scan for each eligible patient, the system forwarded more than 100 duplicate versions of the same scan. These duplicates were sent both to the on-premises AI server and to the cloud, triggering redundant analyses and flooding the workstation with repeat alerts. Despite 12 weeks of troubleshooting involving the hospital PACS team, the external contractor, and Nanox engineers, the underlying cause of the duplication was not resolved. As an interim fix, Nanox developed a custom software patch for this site that blocked forwarding of more than one scan per patient per day, preventing further duplication.

### Delays due to gaps in understanding data architecture

Despite detailed technical requirements for the deployment of HealthVCF (outlined in the “Methods” section), implementation across sites revealed major inconsistencies in documentation and understanding of the data flow architecture. Several Trusts lacked early access to comprehensive information on how patient data would be processed, where de-identification occurred, and which system components required internet connectivity.

Local IG and IT teams often requested additional clarification from the vendor to understand the chain of custody for patient data. For example, while the tool ran on an EU-based server in Ireland, auxiliary services hosted in the United States (eg, software updates and monitoring via AWS) raised questions about data security and compliance, even though no identifiable patient data were transmitted. In some sites, this uncertainty delayed approvals until risk assessments could be completed or additional documentation was provided.

## Discussion

### Variation in IG approval

The equitable implementation of AI solutions within healthcare for patient benefit has been identified as a high priority^23^ but faces numerous challenges,^10^ particularly in the domains of IG assurance and IT infrastructure. In this multi-site study, we identified significant heterogeneity in IG approval processes across NHS trusts, including variation in the number and type of documents required, local interpretations of national frameworks such as DPIA and DTAC with inconsistent expectations around data flows and security protocols. These differences contributed to delays ranging from several months to over a year in some sites. While each Trust must take responsibility for due diligence in the handling of patient data, the lack of standardization leads to inefficiencies, duplication of effort, and in one case, complete withdrawal from the project. This is particularly problematic for vendors and clinical teams operating across multiple sites, who are forced to navigate bespoke and opaque approval landscapes.

### IT infrastructure, deployment bottlenecks, and third-party dependencies

Even when IT services were delivered in-house, sites reported major delays in configuring essential components such as DICOM forwarding and server connectivity. Where radiology services or PACS platforms were outsourced to third-party providers, additional barriers emerged. In one hospital, installation could not proceed until a £20 000 IT support fee had been received by the external supplier. These costs were not anticipated during the early planning phase and highlight the critical importance of mapping out contractual responsibilities and budget expectations when third-party providers are involved. Many radiology departments may underestimate or overlook these dependencies, but they can materially impact project timelines and viability. Although scan routing and RIS/PACS integration are familiar operational tasks, recent increases in data sensitivity, cyber risk, and cloud-based workflows have made these processes slower and more resource-intensive than in previous software implementations.

### Cloud architecture and data sovereignty considerations

The architecture of the AI system used in this study involved both on-premises and cloud components, with cloud servers located in Ireland (EU) and server instances used for support and monitoring based in the United States. While all data transmissions were encrypted using up-to-date security protocols and patient identifiers were not shared, the location of cloud services still triggered scrutiny from local IG teams. Post-Brexit, data sharing between the United Kingdom, EU, and United States is subject to evolving legal interpretations and varying thresholds for risk acceptance. This uncertainty introduced delays in some sites, especially where IG officers were unfamiliar with the specific safeguards provided under GDPR or the UK International Data Transfer Agreement. The geographic location of cloud infrastructure is an often overlooked but increasingly critical consideration in AI deployment, particularly in systems involving any patient-derived data.

In addition to location, data deletion policies were a key area of concern, especially in the absence of clear documentation from some vendors. By contrast, some commercial AI providers such as MVision, a company offering AI tools for radiotherapy planning, adopt explicit policies to enhance trust and accelerate governance approval such as their commitment to automatic deletion of de-identified imaging data from their cloud systems within 24 h of upload.^24^ Including comparable, time-bound deletion protocols as part of AI deployment documentation could help mitigate IG concerns and provide a clearer benchmark for Trusts during the review process.

### Workforce readiness and training gaps

Although some teams had prior exposure to digital innovation projects, many had little or no experience with AI deployment or the specific governance and interoperability requirements that it entails. While there are resources such as the British Institute of Radiology AI Education Essentials videos for clinicians^25^^,^^26^ and the EU HelloAI programme,^27^ there are currently no recognized certificates for training of IG teams in AI with most IG teams relying on in-house expertise and experience with no external standardization. Other training opportunities include an apprenticeship model and advice from the NHS Strategic Information Governance Network.^28^ As with the introduction of PACS, teleradiology, or dose-monitoring tools, sustained investment in upskilling, in line with the Topol Review’s upskilling mandate, will be crucial for AI-augmented services to be embedded equitably across NHS services.

### Pre-existing system constraints

Importantly, the barriers described here are not unique to AI and reflect long-standing structural limitations in the way software is introduced into NHS settings. The tightening of data protection regulations through GDPR and the Caldicott principles, growing security scrutiny following NHS cyberattacks, and the proliferation of software tools all mean that existing processes are under strain. However, what makes AI different is the scale and frequency of innovations now entering clinical care. This volume can increase the burden on local teams and expose the inefficiencies of the current model. While IG and IT diligence are an essential component of AI deployment, the current variations in practice and delays are no longer tenable for AI-based technologies to deliver the expected patient benefit in the healthcare settings.

### Strengths and limitations of the study

A major strength of this research is its real-world setting, with participating hospitals selected irrespective of their AI readiness. While we included hospitals from England and Wales, the findings may not be generalizable to other hospitals in England and Wales as well as Scotland, where there is a single PACS provider, and Northern Ireland. We did include tertiary referral centres and district general hospitals but recognize that studying additional sites may have disclosed other challenges. In addition, the use of AI in the NHS is rapidly evolving, and some trust policies may have changed since the study was initiated, solving some issues but potentially generating others. It is not clear whether any of the participating Trusts had previously deployed AI technologies in radiology or other clinical domains. Furthermore, another limitation was that there was no prospective plan in place to formally capture or measure variables such as the number of emails, the volume and type of documents submitted, staff response times, and meetings to better assess the efficiency of AI deployment. In addition, we were only notified of the time for approval and assurance. Further work should also consider other aspects of workload, such as meeting times, emails, and the number of people involved to better assess the efficiency of AI deployment. Another limitation is that we did not collect the incidental finding rate (ie, proportion of scans with vertebral fractures) at each site prior to AI deployment. Finally, the audits did demonstrate that approximately 50% of VCF on routine CT scans in adults were not reported. The AI-enabled pathway is likely to identify a significant number of discrepancies from the routine radiology report. Further work is needed to identify pragmatic solutions to address this that can be scalable to 1000s of scans per year.

## Conclusion

Overall, this real-world study of integrating an AI tool into radiology systems highlighted specific IG/IT challenges that delayed or prevented deployment. Our experiences from the implementation of AI solutions within the NHS have led to a number of recommendations to improve the effectiveness and efficiency of deployment for local and national leads of radiology, IT, and IG services to consider. This, in turn, supports improved patient outcomes realizing the potential benefits from AI-enabled healthcare delivery.

## Supplementary Material

ubaf017_Supplementary_Data

## Data Availability

The individual site forms are not available due to confidentiality rules.
