# Supplementary material for: Lessons learned from a multi-centre implementation of an artificial intelligence algorithm to detect vertebral fractures for radiology, information technology, information governance and clinical leads
Source: BJR Artif Intell. 2025 Oct 24;2(1):ubaf017. doi: 10.1093/bjrai/ubaf017 (PMC13045679; doi:10.1093/bjrai/ubaf017)
Supplement: ubaf017_Supplementary_Data [file ubaf017_supplementary_data.zip › Supplementary_Table_1_and_caption_for_Figure_1.docx]

**Supplementary Table 1:** Pre-installation IT form for sites to complete

| Medical Facility Information  General information on the Medical Facility | | | | | | | | | | | |
| --- | --- | --- | --- | --- | --- | --- | --- | --- | --- | --- | --- |
| Customer Name | | |  | | | | | | | |  |
| Contact Information | | | | | | | | | | | |
| Role | | | Name | | Email Address | | | | | Cell# | |
|  | | |  | |  | | | | |  | |
|  | | |  | |  | | | | |  | |
| Nanox.AI Forwarder Server Minimal Requirements  The Nanox.AI on-premise requires a Linux server in the hospital network with the following specifications: | | | | | | | | | | | |
| CPU | | | 8 | | | | | | | |  |
| RAM | | | 32GB | | | | | | | |  |
| Hard Disk* | | | 1TB | | | | | | | |  |
| Supported Operations Systems | | | Ubuntu 20.04 / Ubuntu 22.04 / RHEL 8.5 | | | | | | | |  |
| System user should be a root user, named - “zebramed” | | | | | | | | | | | |
| * 900GB disk storage should be allocated to the root partition “/”. 100GB disk storage should be allocated to the root partition “/var/lib” | | | | | | | | | | | |
| Network Requirements  The Nanox.AI on-premise VM requires an internet access with a minimum of 30 Mbps (50 Mbps is optimal) upload speed. This information helps us set an internal configuration to make sure network issues will not affect the system. | | | | | | | | | | | |
| Approximate Download speed  (e.g., 50Mbps) | | |  | | | | | | | |  |
| Approximate Upload speed  (e.g., 50Mbps). | | |  | | | | | | | |  |
| Remote Access  Remote access allows Nanox.AI’s service engineers to maintain and support the server. | | | | | | | | | | |  |
| I understand that Remote Access to Nanox AI engineers is essential for customer support, and it is permitted | | | | | | | Yes No | | | |  |
| Please share the VPN information and credentials through secure channels. | | | | | | | | | | |  |
| Nanox.AI Cloud - Public DNS  The Nanox.AI on-premise requires internet access (HTTPS/443) to the nearest AWS public DNS servers. | | | | | | | | | | | |
| Ireland | | | DNS  ima-cloud-prd-euw1.zebra-med.com  IPs  54.220.92.241  79.125.112.113  52.209.186.219 | | | | | | | |  |
| Public CIDR  The Nanox.AI cloud requires the public CIDR address of the on-premise server so it can be whitelisted on the cloud’s gateway | | | | | | | | | | | |
| Please email to Nanox AI Customer Success the Public CIDR of OPI | | | | | | | | | | |  |
| Security  The Nanox.AI on-premise requires internet access to the following public DNS servers.  (*) The protocol is TCP, TLS (the most updated version available) HTTPS outgoing port 443.  NO PHI is shared on any of the following services. | | | | | | | | | | | |
| K3s Manager* | | | 54.145.24.147  52.71.84.131 | | | | | | | |  |
| Download Server* | | | 18.205.133.102  3.208.247.141 | | | | | | | |  |
| Monitoring & Alerting* | | | 162.247.240.0/22 | | | | | | | |  |
| PACS and Reporting system Information | | | | | | | | | | | |
|  | | | Vendor name | | | | | Version | | |  |
| PACS System | | |  | | | | |  | | |  |
| Reporting System | | |  | | | | |  | | |  |
| DICOM forwarding from PACS to Nanox.AI  The Nanox.AI  on-premise listens to new studies through a DICOM SCP protocol on port 9996, AETitle of Nanox.AI | | | | | | | | | | | |
| Nanox.AI shall provide the on-premise IP address (Port=9996 and AETitle=NANOXAI) | | | | | | | | | | |  |
| Does the PACS require a proxy server? If so, does it require authentication? | | | | | | Yes No Need further explanation | | | | |  |
| Optional Integration types | | | | | | | | | |  |  |
| Secondary Capture - Recipient PACS configuration  Nanox.AI sends Secondary Capture Dicom on suspicious findings to the recipient PACS | | | | | | | | | | | |
| Please choose whether this integration type is applicable or not | | | | | | | Yes No N/A | | | | |
| Host | | | Port | | | | | AETitle | | |  |
|  | | |  | | | | |  | | |  |
| Reviewed and completed By | | | | | | | | | | | |
|  | | Nanox.AI | | | | Customer | | | | | |
| Name | |  | | | |  | | | | | |
| Role | |  | | | |  | | | | | |
| Date | |  | | | |  | | | | | |

**Supplementary Figure 1: Timelines and IT implementation costs by Hospital**

**As separate file**
