# Supplementary figures and images for: Lessons learned from a multi-centre implementation of an artificial intelligence algorithm to detect vertebral fractures for radiology, information technology, information governance and clinical leads
Source: BJR Artif Intell. 2025 Oct 24;2(1):ubaf017. doi: 10.1093/bjrai/ubaf017 (PMC13045679; doi:10.1093/bjrai/ubaf017)

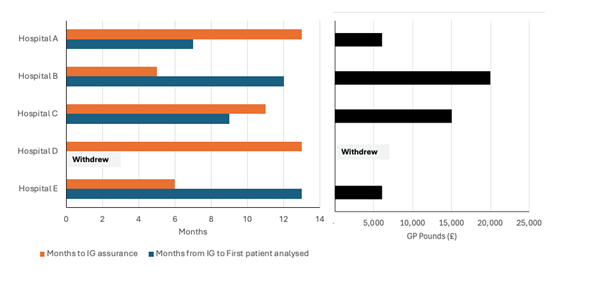

Supplement: ubaf017_Supplementary_Data [file ubaf017_supplementary_data.zip › Supplementary Figure 1.png]
